# Multiparametric MRI: can we assess renal function differently?

**DOI:** 10.1093/ckj/sfae365

**Published:** 2024-11-19

**Authors:** Corentin Tournebize, Maxime Schleef, Aurélie De Mul, Sophie Pacaud, Laurence Derain-Dubourg, Laurent Juillard, Olivier Rouvière, Sandrine Lemoine

**Affiliations:** Service de néphrologie, dialyse, exploration fonctionnelle rénale, Hôpital Edouard Herriot, Hospices Civils de Lyon, Lyon, France; Centre de Référence des Maladies Rares Rénales de la Réunion et du Grand-Est «MaReGe», filière ORKID, Lyon, France; CarMeN Laboratory, Inserm U1060, INRA U1397, Université Claude Bernard Lyon-1, Bron, France; Service de néphrologie, dialyse, exploration fonctionnelle rénale, Hôpital Edouard Herriot, Hospices Civils de Lyon, Lyon, France; Centre de Référence des Maladies Rares Rénales de la Réunion et du Grand-Est «MaReGe», filière ORKID, Lyon, France; CarMeN Laboratory, Inserm U1060, INRA U1397, Université Claude Bernard Lyon-1, Bron, France; Service de néphrologie, dialyse, exploration fonctionnelle rénale, Hôpital Edouard Herriot, Hospices Civils de Lyon, Lyon, France; Centre de Référence des Maladies Rares Rénales de la Réunion et du Grand-Est «MaReGe», filière ORKID, Lyon, France; Service d'Imagerie Urinaire et Vasculaire, Hôpital Edouard Herriot, Hospices Civils de Lyon, Lyon, France; Service de néphrologie, dialyse, exploration fonctionnelle rénale, Hôpital Edouard Herriot, Hospices Civils de Lyon, Lyon, France; Centre de Référence des Maladies Rares Rénales de la Réunion et du Grand-Est «MaReGe», filière ORKID, Lyon, France; Service de néphrologie, dialyse, exploration fonctionnelle rénale, Hôpital Edouard Herriot, Hospices Civils de Lyon, Lyon, France; Centre de Référence des Maladies Rares Rénales de la Réunion et du Grand-Est «MaReGe», filière ORKID, Lyon, France; CarMeN Laboratory, Inserm U1060, INRA U1397, Université Claude Bernard Lyon-1, Bron, France; Service d'Imagerie Urinaire et Vasculaire, Hôpital Edouard Herriot, Hospices Civils de Lyon, Lyon, France; LabTau, INSERM U1052, Université de Lyon, Lyon, France; Service de néphrologie, dialyse, exploration fonctionnelle rénale, Hôpital Edouard Herriot, Hospices Civils de Lyon, Lyon, France; Centre de Référence des Maladies Rares Rénales de la Réunion et du Grand-Est «MaReGe», filière ORKID, Lyon, France; CarMeN Laboratory, Inserm U1060, INRA U1397, Université Claude Bernard Lyon-1, Bron, France

**Keywords:** blood oxygen level-dependent MRI, diffusion-weighted MRI, magnetic resonance relaxometry, multiparametric magnetic resonance imaging, renal function evaluation

## Abstract

We are lacking tools to evaluate renal performance. In this review, we presented the current knowledge and potential future applications in nephrology of new magnetic resonance imaging (MRI) techniques, focusing on diffusion-weighted (DWI) MRI, blood oxygen level-dependent (BOLD) MRI, and magnetic resonance relaxometry (T1 and T2 mapping). These sequences are sensitive to early changes in biological processes such as perfusion, oxygenation, edema, or fibrosis without requiring contrast medium injection and avoids irradiation and nephrotoxicity. Combining these different sequences into the so-called “multiparametric MRI” enables noninvasive, repeated exploration of renal performance on each kidney separately. DWI MRI, which evaluates the movement of water molecules, is a promising tool for noninvasive assessment of interstitial fibrosis and the cortical restricted diffusion has a prognostic value for the deterioration of renal function in diabetic nephropathy. BOLD MRI is sensitive to changes in renal tissue oxygenation based on the paramagnetic properties of deoxyhemoglobin and is of particular interest in the setting of renal artery stenosis to assess tissue oxygenation in the post-stenotic kidney. This sequence can be used for predicting degradation of renal function in chronic kidney diseases (CKD) and might be useful in preclinical studies to assess nephroprotective and nephrotoxic effects of drugs in development. T1 and T2 relaxation times change with tissue water content and might help assessing renal fibrosis. A corticomedullary dedifferentiation in T1 has been observed in CKD and negatively correlates with glomerular filtration rate. Data on the significance of T2 values in renal imaging is more limited. Multiparametric MRI has the potential to provide a better understanding of renal physiology and pathophysiology, a better characterization of renal lesions, an earlier and more sensitive detection of renal disease, and an aid to personalized patient-centered therapeutic decision-making. Further data and clinical trials are needed to allow its routine application in clinical practice.

## INTRODUCTION

We are lacking tools to evaluate renal performance. Serum creatinine level is an indirect marker of glomerular filtration rate (GFR) and increases when a significant filtration capacity has been lost and kidney damage is advanced [1]. Renal fibrosis and oxygen content are also two major determinants of renal performance but are challenging to measure either in experimental set-up or in clinical practice because it requires invasive procedures [2, 3]. New magnetic resonance imaging (MRI) techniques applied to kidney imaging are sensitive to early changes in biological processes such as perfusion, oxygenation, edema, or fibrosis without requiring contrast medium injection, and avoids irradiation. Combining these different sequences into the so-called “multiparametric MRI” enables noninvasive, repeated exploration of renal performance on each kidney separately. In 2017, the European Cooperation in Science and Technology funded the European network of researchers in renal MRI called PARENCHIMA (www.renalmri.org) to harmonize MRI biomarker measurements and improve clinical translation of MRI in chronic kidney diseases (CKD) [4, 5]. In 2018, this multidisciplinary group published four in-depth reviews of the most commonly used renal functional MRI techniques [6–9]. Because of the lack of technical recommendations that hinders comparison between the studies, PARENCHIMA group initiated a drive toward standardization and generated consensus statement to five MRI sequencies [T1 and T2 mapping, arterial spin labeling (ASL), diffusion-weighted imaging (DWI) and blood oxygenation level-dependent (BOLD)] [10–13]. Future work includes major longitudinal multicenter studies such as iBEAT (NCT03716401) and AFiRM (NCT04238299) studies that focus on identification of MRI biomarkers associated with CKD progression, and may include development of renal MRI emerging such as like magnetization transfer imaging, magnetic resonance elastography, MRI spectroscopy, chemical exchange saturation transfer MRI, sodium MRI, or hyperpolarized MRI [14–24].

In this review, we present an overview of the interest and applications in nephrology, and summarize the recent data from the four most-available functional MRI sequencies DWI, BOLD, T1, and T2 mapping. Because of the limited capability of today's scanners to easily perform renal ASL, this sequence was not included in our review [11]. The different sequences and their potential applications are summarized in Table 1.

**Table 1:** Summary of the different sequences and their potential applications.

MRI sequence	Principle	Measurement	Areas of application in nephrology
DWI	Evaluates the movement of water molecules in tissues	Monoexponential: ADC IVIM: water diffusion in tissues (*D*); pseudodiffusion D*; flowing fraction (*F*) DTI: fractional anisotropy (FA) mean diffusivity (MD) independent of anisotropy	• Distinguishment of the different stages of CKD • Correlation with GFR • Detection of fibrosis and chronic lesions (cortical ADC) • Prediction of renal atrophy, fibrosis and inflammation following ischemia-reperfusion injury
BOLD	Measures the amount of deoxyhemoglobin, a reflection of tissue deoxygenation	R2* (1/s)/T2* (s)	• Reflection of tissue oxygenation • Assessment of the severity of RAS • Prediction of renal function deterioration (cortical R2*) • Detection of tubular dysfunction (Furosemide test)
T1 mapping	Longitudinal relaxation time	T1 (ms)	• Cortical T1: correlation with GFR • Cortical T1 and ΔT1: detection of fibrosis • Detection of edema and inflammation in various kidney compartments • Additional data required
T2 mapping	Transversal relaxation time	T2 (ms)	• Detection of edema and inflammation in the various compartments of the kidney, of interest in ischemia-reperfusion lesions • Early assessment of polycystic kidney disease • Additional data required

ΔT1, cortico-medullary difference.

## DIFFUSION-WEIGHTED MAGNETIC RESONANCE IMAGING

### Biophysical and technical principles

DWI MRI evaluates and highlights the differences in the mobility of water molecules in tissues, including Brownian motion (random movement of a particle immersed in a fluid, subject to no other interaction) [7]. DWI is achieved by applying diffusion gradients on either side of a 180° radiofrequency pulse, usually during a T2-weighted echo-planar imaging (EPI) sequence. When water molecules are mobile and diffuse rapidly, their spins cannot be rephased by the rephasing pulse, resulting in a reduced signal. It is necessary to apply diffusion gradients in several spatial directions. The *b*-value (the degree of diffusion weighting applied) is a factor that reflects the strength and timing of the gradients used to generate diffusion*-*weighted images. In renal imaging, *b*-values ≤800 s/mm² are recommended [12]. DWI MRI is widely used in neuroradiology. Its application in renal imaging is expanding.

**Figure 1:**
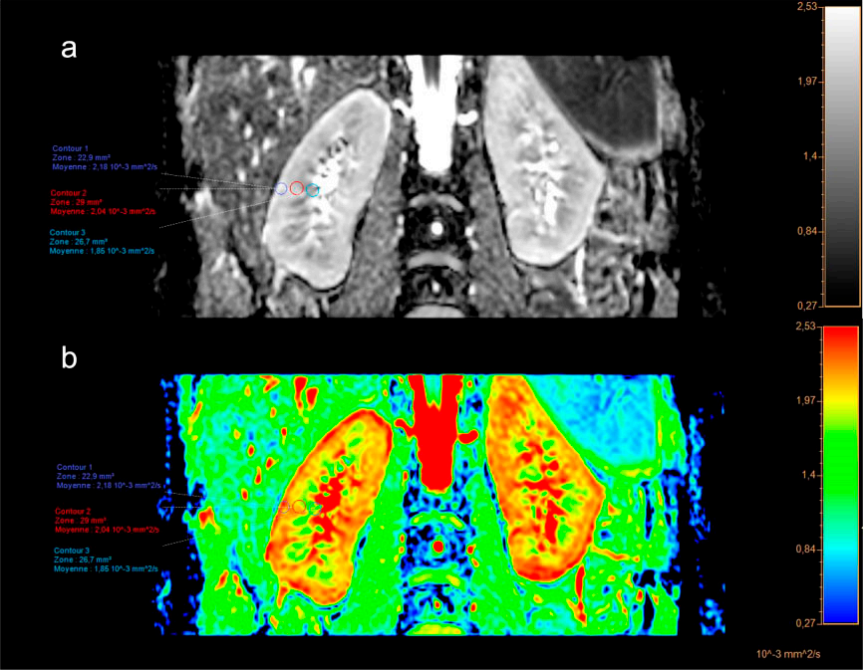
Coronal DWI representative image (monoexponential model) with ADC map in a 30-year-old healthy subject showing the corticomedullary differentiation. (**a**) ADC map. ADC values are measured in three ROI: the cortex (navy blue circle), the outer medulla (red circle), and the inner medulla (sky blue circle). Results show a decrease of ADC values from 2.18 × 10^−3^ mm²/s in the cortex to 1.85 × 10^−3^ mm²/s in the inner medulla (**b**) ADC map colorized. The cortical ADC values are physiologically significantly higher (red) than the medullary ADC values (yellow). Acquisition parameters: voxel volume: 1.8 × 1.8 × 3 mm; repetition time: 3000 ms; echo time: 70 ms; matrix: 156 × 67; field of view: 280 × 120; acquisition time: 135 s; *b*-values: 0, 800.

The DWI signal decreases as the *b*-value of the diffusion gradients increases. The apparent diffusion coefficient (ADC) is classically calculated by considering the DWI signal decay as a monoexponential equation (Fig. 1). However, blood microcirculation and tubular flow also impact the decrease of the signal, especially for low *b*-values, resulting in a bi-exponential decay. The so-called intravoxel incoherent motion (IVIM) model considers this bi-exponential decay to compute a true diffusion coefficient (*D*), a pseudodiffusion coefficient (D*), and the flowing fraction (*f*) resulting from microcirculation within blood vessels and tubules. Although the IVIM parameters better reflect the underlying physiological processes, they are limited by their sensitivity to noise. The monoexponential model remains the most used because of its easier application.

Finally, it is possible to encode the diffusion signal in a great number of directions to assess the spatial dependency of water diffusion. This so-called diffusion tensor imaging (DTI) can assess tissue micro-architecture and its degree of anisotropy.

### Applications

#### Assessment of fibrosis in chronic kidney disease

DWI MRI is a promising tool for noninvasive assessment of interstitial fibrosis (IF) for which the gold standard remains an invasive kidney biopsy procedure. In animal models, a negative correlation between cortical or kidney ADC value and fibrosis has been shown [25–27]. IVIM and DTI allow to assess fibrosis more accurately than ADC [28–33].

In humans, several studies similarly showed a negative correlation between cortical, corticomedullary ADC difference, or kidney ADC and histological data such as IF percentage, glomerulosclerosis, and tubular atrophy [34–37]. In 22 CKD patients (mean eGFR 39 ± 14 ml/min/1.73 m²), cortical ADC and corticomedullary differentiation were significantly altered in patients reaching a threshold of >40% of IF [38]. The combination of cortical ADC and T1 mapping could also improve IF quantification as compared to each sequence individually [37, 39]. Clinical uses of IVIM or DTI were also consistent with animal studies, with an accurate detection of IF [40–44]. A negative correlation between fractional anisotropy measured in DTI and the percentage of glomerulosclerosis or IF was observed in two series of 51 and 75 CKD patients, respectively [45, 46]. Key studies about the use of DWI MRI in IF assessment in humans are described in Table 2.

**Table 2:** Key data on the use of DWI MRI in the assessment of renal fibrosis in human.

Study	Participants	Estimated GFR (ml/min/1.73 m²)	Magnetic field strength (T)	Diffusion model and technique	*b*-values (s/mm²)	Cortical ADC (10^−3^ mm²/s)	Medullary ADC (10^−3^ mm²/s)	Kidney ADC (10^−3^ mm²/s)	Main result
Inoue, *J Am Soc Nephrol* (2011) [34]	*n* = 142, including 76 non-diabetic CKD patients (Group 1), 43 patients with diabetic nephropathy (Group 2), 23 patients with AKI (Group 3)	Group 1: 45.8 ± 30.1 Group 2: 43.8 ± 27 Group 3: 16.0 ± 10.2	1.5	Monoexponential	0, 50, 100, 150, 300, 500, 750, 1000	NA	NA	NA	ADC and T2* values are used to assess renal tubulointerstitial alterations and cortical hypoxia, respectively.
Zhao, *Clin Radiology* (2014) [36]	*n* = 70, including 40 CKD (Biopsy performed in 25 patients: 6 MN, 5 MCD, 5 atypical MN, 5 IgA nephropathies, 3 proliferative mesangial glomerulonephritis, 1 hypertensive nephropathy) and 30 controls	NA	3	Monoexponential	0, 800	2.41 ± 0.33	2.17 ± 0.34	NA	Strong correlation between ADC values and fibrosis.
Liu, *Eur Radiol* (2015) [45]	*n* = 51, including 5 MCD, 12 IgA nephropathies, 20 MN, 1 crescentic necrotizing glomerulonephritis, 5 lupus nephritis, 2 HBV-associated glomerulonephritis, 1 hypertensive nephropathy, 2 FSGS, 3 rheumatoid purpuras	NA	3	Monoexponential, DTI	0, 400, 600	2.16 ± 0.29	2.12 ± 0.28	NA	FA measured in DTI is correlated with fibrosis and renal function.
Feng, *Eur Radiol* (2015) [46]	*n* = 95, including 75 CKD (classified from stage 1 to 5, with 15 patients in each class) and 20 controls	114 ± 7.36 (controls) to 8.53 ± 2.88 (CKD Stage 5)	1.5	Monoexponential, DTI	0, 600	NA	NA	Controls: 2.33 ± 0.03 Stage 1: 2.31 ± 0.03 Stage 2: 2.15 ± 0.04 Stage 3: 2.04 ± 0.03 Stage 4: 1.96 ± 0.04 Stage 5: 1.86 ± 0.03	ADC and FA values are correlated with the severity of CKD, the percentage of glomerulosclerosis and the amount of interstitial fibrosis.
Friedli, *Sci* *Rep* (2016) [27]	*n* = 33 kidney allograft recipients	50 ± 22	3	Monoexponential	0, 10, 20, 40, 60, 150, 300, 500, 700, 900	From 1.634 to 2.816	from 1.735 to 2.620	NA	Corticomedullary ADC difference was negative in all allografts harboring >40% fibrosis and positive in allografts with <40% fibrosis. Corticomedullary ADC difference outperformed corticomedullary T1 difference with a stronger negative correlation to fibrosis.
Xu, *Abdom Radiol* (2018) [35]	undergoing kidney biopsy	NA	1.5	Monoexponential	0, 500	NA	NA	Absence of tubular atrophy: 2.51 ± 0.31 Mild tubular atrophy: 2.44 ± 0.23 Moderate tubular atrophy: 2.33 ± 0.20 Severe tubular atrophy: 2.10 ± 0.12	Negative correlation between ADC values and severity of tubular atrophy and interstitial fibrosis.
Berchtold, *NDT* (2020) [37]	*n* = 164 CKD patients undergoing kidney biopsy for clinical reasons including 118 kidney allograft patients and 46 native kidney patients	Total: 57.2 ± 24.2 CKD: 59.2 ± 33.7 Allograft: 56.4± 19.4	3	Monoexponential	0, 50, 100, 150, 200, 250, 300, 500, 700, 900	NA	NA	NA	Corticomedullary ADC difference is better correlated to interstitial fibrosis than absolute cortical or medullary ADC values and allowed detection of extensive fibrosis with good specificity.
Buchanan, *NDT* (2020) [38]	*n* = 44, including 22 CKD (11 glomerular nephropathies, 3 tubulointerstitial nephropathies and 8 ischemic nephropathies) and 22 controls	CKD: 39 ± 14 Controls: 92 ± 12	3	IVIM	0, 5, 10, 20, 30, 50, 100, 200, 300, 400, 500	CKD: 1.94 ± 0.44 Controls: 2.3 ± 0.1	CKD: 2.03 ± 0.22 Controls: 2.2 ± 0.1	NA	cortical ADC and corticomedullary differentiation in ADC can detect patients with >40% interstitial fibrosis.
Berchtold, *Kidney Int* (2022) [37]	*n* = 197 patients undergoing kidney biopsy for clinical reasons including 43 patients with CKD and 154 patients with a kidney allograft	CKD: 55.1 ± 33.4 Allograft: 53.4 ± 20.7	3	Monoexponential	0, 50, 100, 150, 200, 250, 300, 500, 700, 900	NA	NA	NA	Low and negative corticomedullary ADC difference is a predictor of renal function decline and dialysis initiation in CKD and kidney allograft patients, independent of baseline renal function and proteinuria.
Zhu, *Magn Reson Imag* (2023) [42]	n = 89, including 79 CKD (22 IgA nephropathies, 21 MN, 13 diabetic nephropathies, 4 lupus nephritis, 4 MCD, 15 others), classified into 2 groups according to glomerulosclerosis and interstitial fibrosis scores: • Group 1: Mild histological lesions • Group 2: Moderate to severe histological lesions • 10 controls	CKD: 89.34 (70.02–106.20) Controls: 102.2 (98–116)	3	Monoexponential, IVIM, DTI	0, 25, 50, 100, 150, 200, 400, 800, 1000	Controls: 1.98 ± 0.17 Group 1: 1.94 ± 0.15 Group 2: 1.94 ± 0.34	Controls: 1.9 ± 0.18 Group 1: 1.87 ± 0.13 Group 2: 1.87 ± 0.23	NA	IVIM and DTI findings make it possible to differentiate the histological grades of fibrosis. No correlation found between ADC values and the degree of histological lesions.
Mao, *Curr Med Imaging* (2023) [43]	*n* = 100, including 80 IgA nephropathies (divided into 2 groups according to histological score) and 20 controls	NA	1.5	Monoexponential, IVIM	0, 25, 50, 80, 100, 150, 300, 500, 800, 1000	NA	NA	Moderate to severe histological involvement: 1.71 ± 0.12 Mild histological involvement: 1.84 ± 0.10 Controls: 1.84 ± 0.10	Diffusion parameters are significantly lower in moderate to severe histological damage compared with mild histological damage and healthy subjects. IVIM is better than monoexponential model for assessing histological lesions in IgA nephropathy.

NA, non-applicable; MN, membranous nephropathy; MCD, minimal change disease; FSGS, focal segmental glomerulosclerosis. Data are expressed as mean ± standard deviation.

#### Association with deterioration of renal function in chronic kidney disease

Most studies evaluating DWI MRI in CKD found a positive correlation between ADC and GFR, and a negative correlation with proteinuria, a biomarker of CKD progression [38, 45, 47–53]. Similar results were found with IVIM and DTI [45, 48]. DWI MRI allows to distinguish kidneys of healthy subjects from those of CKD patients, and even to classify them according to their CKD stages [51, 52, 54–59]. The use of DWI MRI enables sensitive detection of early stages of diabetic nephropathy, earlier than microalbuminuria [60–63], and cortical D* measured in IVIM was identified as an independent marker to exclude diabetic nephropathy alongside with the absence of diabetic retinopathy in a cohort of 63 type 2 diabetic patients [64].

DWI MRI also has prognostic value as cortical ADC predicted the deterioration of renal function in diabetic nephropathy [65]. In addition, in a series of 43 CKD patients and 154 renal transplant recipients, those with an initial ADC corticomedullary dedifferentiation were 4.62 times more likely to experience rapid deterioration in renal function or to require dialysis (median follow-up of 2.2 years), irrespective of initial baseline renal function or proteinuria [66].

#### Acute kidney injury diagnosis

According to preclinical studies and pathophysiology of renal ischemia-reperfusion injuries (IRI), DWI is of particular interest for two timings. In a 60 min unilateral IRI rodent model, early histological changes up to 7 days after reperfusion composed of interstitial edema, tubular changes and inflammatory cell infiltration led to a decrease in ADC values in the cortex, outer and inner medulla [67]. In addition, in a moderate (clamp time 35 min) and severe (clamp time 45 min) unilateral IRI murine model, Hueper *et al.* showed an early and significant decrease in ADC values, with maximal changes seen at day 7, which was associated with the severity of acute kidney injury (AKI) [68]. At later time points, 28 days after reperfusion, ADC reduction was associated with inflammatory cell infiltration, IF, and loss of kidney volume. Additional studies, including histological analysis, and other AKI models are needed to confirm these results and support clinical translation. In humans, only one study with histopathological analysis available was conducted, including four patients diagnosed with acute interstitial nephritis and showed a decrease in ADC that was not associated with histological lesions [69]. When comparing 10 AKI vs 9 non-AKI patients without prior CKD treated in intensive care for respiratory failure due to COVID-19, Luther *et al.* did not observe difference in DWI measures, but MRI was only performed at a median of 1 day from AKI onset, AKI were mostly stage 1, and no histological analysis was done [70]. Conversely, DWI MRI was performed 14 after lung-transplant surgery in 54 patients, showing a reduction in ADC in patients who presented AKI, but not in those without AKI [71]. Additional studies suggested that DWI might be a tool to early assess AKI in severe acute pancreatitis and contrast-induced nephropathy [72, 73]. Once again, the absence of histological analysis and the different timing of evaluation hinders interpretation of the results.

## BLOOD OXYGENATION LEVEL-DEPENDENT MAGNETIC RESONANCE IMAGING

### Biophysical and technical principles

**Figure 2:**
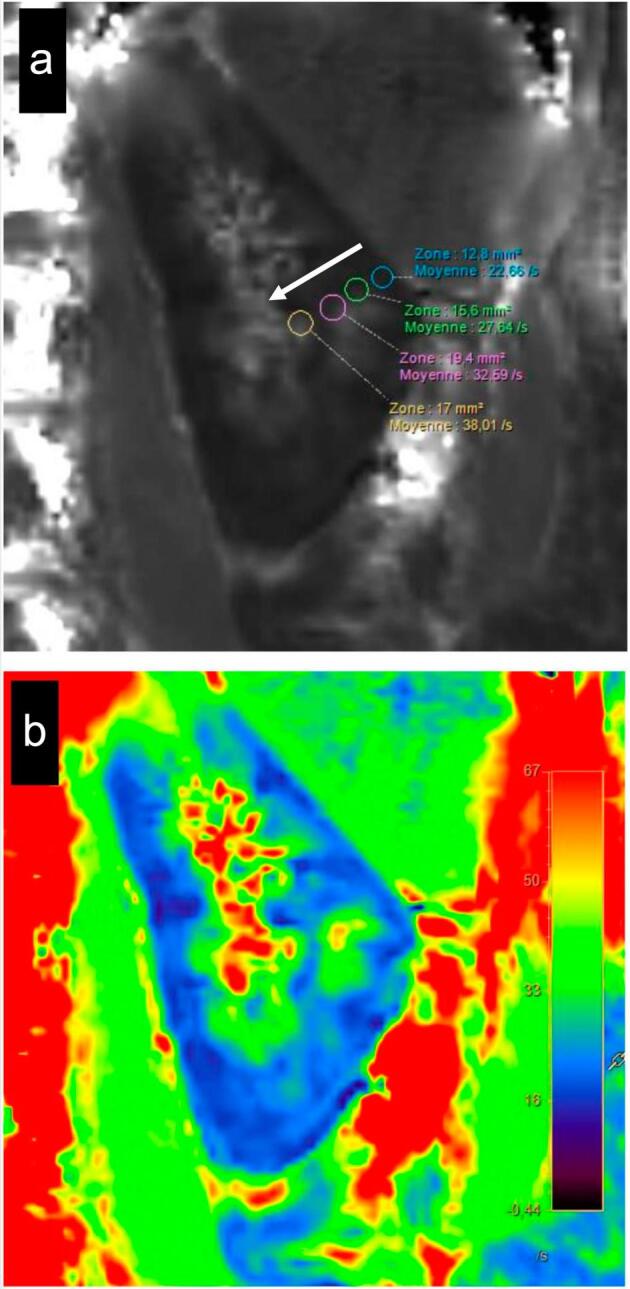
Representative image of left kidney corticomedullary gradient measured with BOLD MRI in a 30-year-old healthy man. (**a**) BOLD MRI map showing the corticomedullary gradient (white arrow). R2* values are measured in four ROI: the cortex (blue circle), the outer medulla (green circle), the medulla (purple circle), and the inner medulla (yellow circle). Results show an increase in R2* values from 22.66 s^−1^ in the cortex to 38.01 s^−1^ in the inner medulla consistent with the known physiologically relative medullary hypoxia. (**b**) Colored BOLD MRI. R2* values are higher (red) in the inner medulla than in the cortex (blue). Acquisition parameters: voxel volume 1.9 × 1.9 × 6 mm; repetition time: 120 ms; echo time 4.3 ms; matrix: 184 × 145; field of view: 345 × 276; acquisition time 18 s. ROI were placed manually.

BOLD MRI is sensitive to changes in tissue oxygenation based on the paramagnetic properties of deoxyhemoglobin. Red blood cells oxygenated contain oxyhemoglobin, which is diamagnetic, i.e. it acquires a very weak magnetization in the opposite direction to the magnetic field and is non-active in MRI. Deoxygenated red blood cells contain deoxyhemoglobin, which is paramagnetic, meaning that under the effect of a magnetic field, it acquires a magnetization directed in the same direction as the external magnetic field. This phenomenon is detectable in MRI and results in an increase in the apparent relaxation rate R2*, expressed in s^−1^ (the inverse of the apparent spin relaxation time T2*). Therefore, a decrease in R2* indicates a drop in deoxyhemoglobin level and improved tissue oxygenation (Fig. 2). BOLD MRI was validated in pigs in comparison with renal oxygenation directly measured with a microelectrode [74–76]. In 1996, Prasad *et al.* observed a decrease in medullary R2* in seven healthy volunteers after furosemide administration, consistent with preliminary observations in pigs, due to an increase in medullary partial pressure of oxygen via the reduction in activity and therefore oxygen consumption of active tubular transport induced by loop diuretics [77, 78]. These data have been shown to be reproducible [79–83].

BOLD MRI mainly uses single shot EPI and gradient-echo acquisition methods. Acquisitions must be made under breath-hold, as the sequence is prone to motion artifacts. EPI is the fastest acquisition method, reducing motion artifacts, but spatial resolution is limited because the sequence is sensitive to magnetic susceptibility artifacts (distortion of the B0 magnetic field at the interface between two tissues with different magnetic susceptibilities). R2* increases with magnetic field intensity, with medullary R2* 1.5 to 2 times higher in a healthy subject at 3.0 T than with 1.5 T, enabling better corticomedullary differentiation and a better signal-to-noise ratio [79]. Other factors should be taken into account and standardized for BOLD signal interpretation such as blood pH, body temperature, and hematocrit that affect the oxygen dissociation curve, hydration status that can influence tubular volume and the local deoxyhemoglobin volume fraction, glycemia as acute hyperglycemia decreases R2* signal in healthy humans, daytime, and the analysis method [10, 84, 85]. Indeed, most studies have used the manually defined regions of interest (ROI) technique for analysis, with small circles containing a collection of voxels placed manually in the cortex and medulla of each slice. Nevertheless, ROI placement is highly observer-dependent in advanced CKD because of the corticomedullary dedifferentiation [86]. A semi-automated analysis method called twelve-layer concentric objects (TLCO) has been shown to be more objective and reproducible especially for CKD [81, 87]. TLCO requires the operator to define the outer limits of the kidney, then the software divides the selection into 12 concentric layers of equal thickness that constitute ROI. However, the consensus-based technical recommendations for clinical translation of renal BOLD MRI stipulated that, until automated segmentation becomes routinely available, manual segmentation may be preferred [10].

### Applications

#### Renal artery stenosis

BOLD MRI is of particular interest in the setting of renal artery stenosis (RAS) to assess tissue oxygenation in the post-stenotic kidney [88]. Reduced renal blood flow (RBF) leads to changes in renal oxygenation, which could be detected by BOLD MRI in pigs [76, 89, 90]. Juillard *et al.* showed a progressive increase of cortical and medullary R2* as degree of occlusion increased in a unilateral pig RAS model [76]. In humans, cortical and medullary R2* remained preserved in moderate RAS (<60% occlusion) despite reduced RBF and GFR [91]. The expected response to furosemide, i.e. decrease in medullary R2*, is attenuated in patients with RAS, suggesting an alteration in tubular active transport [91–93]. In hemodynamically “severe” RAS (>60% occlusion, peak systolic velocity >384 cm s^−1^, reduced renal function and/or reduced renal volume), cortical, and medullary R2* increased indicating altered oxygenation [92, 94–97]. Rather than using cortical or medullary R2*, Saad *et al.* recommended using fractional tissue hypoxia (percentage of the entire axial image section with R2* > 30 s^−1^), which correlated directly with reduced RBF and GFR [98]. After percutaneous transluminal renal angioplasty (PTRA), R2* decreased and fractional tissue hypoxia improved without being associated with an improvement in GFR, cytokine inflammatory profile and tubulointerstitial damage biomarkers [99]. Therefore, BOLD MRI needs to be coupled with other biomarkers and tools to discuss a revascularization procedure [96, 100, 101].

BOLD MRI has also been used to evaluate therapeutic interventions in RAS. For example, the addition of mesenchymal stem cells to PTRA normalized oxygen-dependent tubular response to furosemide [102]. Similarly, the use of Elamipretide (a mitochondria-targeting agent) in combination with PTRA led to an increase in fractional hypoxia after treatment [103].

In summary, severe RAS is characterized on BOLD MRI by an increase in R2* and/or abolition of the response to furosemide, and BOLD MRI might be a tool that can be integrated into the therapeutic discussion although further data are still needed to determine its clinical relevance. Table 3 describes the key data about the use of BOLD MRI in RAS.

**Table 3:** Key data on the use of BOLD MRI in RAS in human.

Study	Objective	Participants	Age (years)	Average GFR (ml/min/1.73 m^2^)	Magnetic field	Furosemide	R2* cortex (1/s)	R2* medulla (1/s)	Main results
Textor, *J Am Soc Nephrol* (2008) [93]	Assessing tissue oxygenation in RAS	*n* = 26, including 15 RAS patients and 11 controls (HBP)	69 ± 12.5	43	1.5	Yes, 20 mg IV	RAS: 16.7 ± 12.8 HBP: 22.2 ± 2.8	SAR: 16.9 ± 1.3 HBP: 24.9 ± 3.8	Decreased medullary and cortical R2* after furosemide in healthy subjects and in RAS with normal-sized downstream kidneys. Atrophied kidneys downstream of RAS have low R2* levels non-modified by furosemide administration.
Gloviczki, *Hypertension* (2011) [92]	Compare tissue oxygenation and RBF in moderate and severe RAS	*n* = 54, including 13 “moderate” RAS, 17 “severe” RAS and 24 controls (HBP)	65.5 ± 2.8	HBP: 80 ± 19.6 Moderate RAS: 68.4 ± 23.4 Severe RAS: 48.1 ± 20.6	3	Yes, 20 mg IV	HBP: 17.8 ± 2.3 Moderate RAS: 15.7 ± 2.1 and CLK: 16.9 ± 2.1 Severe RAS: 21.6 ± 9.4 and CLK: 17.7 ± 2.7	HBP: 36.8 ± 6.3 Moderate RAS: 37.8 ± 5.7 and CLK: 40.8 ± 5.7 Severe RAS: 39.1 ± 11.8 and CLK: 40.4 ± 8.6	R2* values significantly higher in severe RAS than in moderate RAS and hypertension.
Chrysochou, *NDT* (2012) [101]	Assessing the value of the R2*/isoSK-GFR ratio in predicting revascularization outcomes	*n* = 28, including 16 revascularized RAS and 12 controls	Revascularization: 67 ± 9.4 controls: 55 ± 14.1	Revascularization: 51.6 ± 23.8 Controls 47.7 ± 23.0	3	No	NA ROIs included both cortex and medulla	NA ROIs included both cortex and medulla	The initial R2*/isoSK-GFR ratio was significantly higher in kidneys whose renal function improved after revascularization compared with stable, deteriorated or control renal function.
Saad, *Radiology* (2013) [98]	Evaluate the value of fractional tissue hypoxia rather than manual ROI selection for assessing tissue hypoxia in RAS	*n* = 72, including 40 RAS and 32 controls (HBP)	68 ± 8.8	NA Creatinine (mg/dl): HBP: 0.96 ± 0.26 SAR: 1.3 ± 0.4 Single kidney GFR: HBP: 44.7 ± 12 RAS: 27.7 ± 14.5 and CLK: 42.4 ± 17	3	Yes, 20 mg IV	RAS: wide ROI: 20.5 ± 3.6, small ROI: 18.9 ± 3.6 FH (%): 17.4 ± 11.8 CLK: FH (%): 7.6 ± 4.2, large ROI: 17.6 ± 1.7, Small ROI:17.6 ± 1.7 HBP: FH (%): 9.6 ± 6.4, large ROI: 18.5 ± 2.3 small ROI: 18.5 ± 2.6	RAS: 33.6 ± 7.9 CLK: 36.9 ± 8.7 HBP: 37.2 ± 6.3	Although cortical R2* did not differ, FH was significantly higher in stenotic kidneys than in kidneys with HBP and contralateral kidneys. FH correlates directly with reduced RBF and GFR.
Saad, *Circ Cardiovasc Interv* (2013) [99]	Evaluating the effect of RAS revascularization on hypoxia and markers of renal injury	*n* = 49, including 17 revascularized RAS and 32 controls (HBP)	68 ± 8.8	RAS: 65.6 ± 31.9 HBP: 87.9 ± 24.3	3	Yes, 20 mg IV	RAS: Baseline: 21 ± 4.4 FH (%): 19.2(9.2,28.3) At 3 months: 19.7 ± 2.6 FH (%): 11.5 (5.5,16.5) CLK: Baseline: 18 ± 2.3 FH (%): 14.3 (3.7, 15.4) At 3 months: 19.3 ± 2.8 FH (%): 9.3 (4.6, 20) HBP: 18,5 ± 2.7 FH (%): 8.7 (3.5, 13.3)	ND	Despite reversal of renal hypoxia and partial restoration of RBF after revascularization, renal injury biomarkers and GFR did not improve.
Herrmann, *Clin J Am Soc Nephrol* (2016) [96]	Evaluate associations of tissue oxygenation with downstream kidney function and inflammatory biomarkers in patients with revascularized unilateral RAS compared with medical therapy alone	*n* = 62, including 10 revascularized RAS, 20 medically treated RAS and 32 controls (HBP)	Revascularized RAS: 65.1 ± 9.4 RAS treated medically: 67.5 ± 8.3 HBP: 63.1 ± 16.3	Revascularized SAR: 74 ± 34.8 SAR treated medically: 62.6 ± 21.8 HBP: 87.9 ± 24.3	3	No	Revascularized RAS: Baseline: 21.6 ± 6 FH (%): 22.1 ± 20 At 3 months: 19.8 ± 3.1 FH (%): 14.9 ± 18.3 CLK: 18.4 ± 3.2 FH (%): 10.8 ± 6 At 3 months: 19.2 ± 3 FH (%): 10.7 ± 7.1 Medically treated SAR: 19.8 ± 3 FH (%): 14.1 ± 12.6 At 3 months: 20.7 ± 5 FH (%): 15.2 ± 17.2	ND	Fractional tissue hypoxia decreased after revascularization, and GFR of stenotic kidneys improved at 3 months after revascularization, as compared with medical treatment.
Lal, *Abdom Radiol* (2022) [94]	Compare the R2* value of the post-stenotic kidney with contralateral kidney and controls	*n* = 116, including 65 RAS, 31 HBP and 20 controls	RAS: 40 ± 14 HBP: 36 ± 14 Healthy subjects: 28 ± 4	NA Creatinine (mg/dl): SAR: 2.13 ± 1.34 HBP: 1.02 ± 0.07 Controls: 0.9 ± 0.22	3	Yes, 20 mg IV	RAS: 23.30 ± 5.18 CLK: 21.35 ± 3.77 HBP: 22.18 ± 5.18 Controls:22.63 ± 3.58	RAS: 25.32 ± 5.11 CLK: 24.29 ± 4.43 HBP: 26.82 ± 5.46 Controls: 30.6 ± 4.19	No difference in cortical R2* between the groups. Response to furosemide was reduced in RAS compared with other groups. Increased cortical R2* values and reduced response to furosemide were observed only in atrophic, dedifferentiated kidneys.
Long Zhao, *PloS ONE* (2022) [95]	Comparison of R2* values between RAS and healthy subjects and between different degrees of stenosis	*n* = 70, including 40 RAS and 30 controls	57.63 ± 15.42	RAS:72.03 ± 12.7 Controls: 74.6 ± 12.2	3	No	RAS: 21.14 ± 4.90 Controls: 18.23 ± 1.77	RAS: 36.25 ± 8.04 Controls: 29.61 ± 2.26	Cortical and medullary R2* values were significantly higher in RAS than in healthy subjects. Medullary R2* values were significantly higher in severe stenosis compared to healthy subjects, mild stenosis, and moderate stenosis.

NA, non-applicable; CLK, contralateral kidney; HBP, high blood pressure; FH, fractional hypoxia = percentage of the entire axial image section with an R2* greater than 30 s^−1^; R2*/isoSK-GFR, ratio of R2* to single kidney GFR and renal parenchymal volume measured by radioisotope method coupled with renal scintigraphy; data are expressed as mean ± standard deviation.

#### Chronic kidney disease

BOLD MRI can be used for predicting degradation of renal function in CKD rather than for assessing fibrosis. Increased R2* (or decreased T2*) were shown to be a predictive marker of worsening renal function, independently of other known predictors of CKD progression such as proteinuria [104–107]. By contrast, data concerning the ability of BOLD MRI to diagnose CKD is discordant. Some studies reported an increase in cortical or whole kidney R2* in CKD [81, 108–114], and a corticomedullary dedifferentiation only in moderate to severe CKD [113], while others showed no association [38, 115, 116]. One possible explanation for these discrepancies is that renal pO_2_ can remain stable in the face of changes in renal RBF [117]. Indeed, an increase in total RBF leads to a proportional increase in GFR that stimulates tubular sodium reabsorption and thus a compensative increase in O_2_ consumption. Conversely, lower RBF and GFR lead to a decrease in active tubular reabsorption, theoretically making normal R2* values possible in CKD [118]. Other possible explanations may be the absence of standardized patient preparation, and the use of 1.5 T MRI in some studies. Key data on BOLD MRI in CKD are described in Table 4.

**Table 4:** Key data on BOLD MRI in chronic kidney disease in human.

Study	Objective	Participants	Age (years)	GFR (ml/min/1.73 m^2^)	Magnetic field	R2* cortex (1/s)	R2* medulla (1/s)	R2* total (1/s)	Main results
Michaely, *Kidney Int* (2012) [115]	Evaluate the impact of renal function on the BOLD signal	*n* = 343, including 280 with available estimated GFR	59.0 ± 15.9	NA	1.5 and 3	12.4 ± 2.3 (1.5T) 18.7 ± 5.4 (3T)	21.2 ± 4.9 (1.5T) 32.0 ± 7.7 (3T)	NA	No correlation between R2* values and GFR
Yin, *Eur J Radiol* (2012) [150]	Evaluate the renal oxygenation in T2D by BOLD MRI	*n* = 115, including 48 T2D divided into: 14 simple diabetes, 7 Stages III, 15 stages IV and 12 stages V of diabetic nephropathy according with the Mogensen diabetic nephropathy diagnostic criteria; and 67 controls	Simple diabetes: 52.42 ± 14.91 Stage III: 60.29 ± 10.59; Stage IV: 56.87 ± 12.6 Stage V: 64.75 ± 6.74 Controls: 51.04 ± 13.7	Simple diabetes: 92.68 ± 9.58 Stage III: 88.45 ± 22.29 Stage IV: 55.51 ± 17.38 Stage V: 18.40 ± 10.03 Controls: NA	3	NA	NA	NA	Hypoxia in medulla is more severe and appears earlier than in cortex in diabetic nephropathy. During the progression of diabetic nephropathy, a reversion of corticomedullary oxygenation gradient can be detected
Li, *J Transl Med* (2014) [151]	Investigate the utility of BOLD MRI in the assessment of renal involvement and pathological changes in patients with lupus nephritis	*n* = 81, including 65 lupus nephritis and 16 controls	Lupus nephritis: 34 ± 12 Controls: 35 ± 11	NA	1.5	Lupus nephritis: 11.03 ± 1.60 Controls: 12.63 ± 1.40	Lupus nephritis: 14.05 ± 3.38 Controls: 18.14 ± 2.51	NA	R2* values of the renal cortex and medulla in lupus nephritis were significantly lower than that in volunteers. After treatment in lupus nephritis, higher cortical and medullary R2* values are associated with complete remission
Prujim, *Kidney Int* (2018) [104]	Assess whether renal tissue oxygenation measured by BOLD MRI is associated with impaired renal function at 3 years	*n* = 226, including 120 CKD, 62 HBP and 44 controls	CKD: 56 ± 14 HBP: 56 ± 11 Controls: 47 ± 11	CKD: 55 ± 29 HBP: 90 ± 15 Controls: 97 ± 14	3	CKD: 21.2 ± 3.1 HBP: 20.6 ± 1.7 Controls: 20.4 ± 2.5	CKD: 24.2 ± 2.6 HBP: 24.4 ± 1.8 Controls: 24.6 ± 2.1	NA	Low cortical oxygenation measured on BOLD MRI is an independent predictor of impaired renal function
Shi*, J Int Med Res* (2018) [152]	Determine whether BOLD MRI can contribute to the diagnosis of renal pathological patterns in lupus nephritis	*n* = 12, including 12 lupus nephritis	11.54 (range, 15–52)	100.5	3	NA	NA	NA	BOLD textural analysis using an algorithm based on the gray-level co-occurrence matrix has made it possible to distinguish the different classes of lupus nephritis
Li, *Abdom Radiol* (2019) [113]	Assess the application of BOLD MRI in CKD classification	*n* = 56, including 13 mild CKD, 16 moderate to severe CKD and 27 controls	CKD: 42 Controls: 39	CKD: 118.13 to 9.73 Controls: 110.79 ± 11.17	3	Mild CKD: 16.15 ± 1.10 Moderate to severe CKD: 16.77 ± 1.09 Controls: 13.15 ± 0.74	Mild CKD: 25.45 ± 3.63 Moderate to severe CKD: 18.03 ± 3.15 Controls: 32.62 ± 4.26	NA	Significant difference in cortical and medullary R2* values between the groups. Positive correlation between GFR and medullary R2* values
Sugiyama, *NDT* (2020) [106]	Evaluate the relationship between T2* values in BOLD MRI and ADC values in DW-MRI, and the evolution of renal function over 5 years in CKD patients	*n* = 91, including 91 CKD	55.8 ± 15.6	49.2 ± 28.9	1.5	NA	NA	NA T2*: 74.2 ± 8.5	Decreased oxygenation, characterized by low T2* values, is an independent predictor of CKD progression
Feng, *Br J Radiol* (2020) [153]	Assess the capability of quantitative BOLD imaging to detect early diabetic nephropathy	*n* = 45, including 30 T2D, divided in 15 moderately increased albuminuria (MAU) and 15 normal to mildly increased albuminuria (NAU); and 15 controls	NAU: 52.33 ± 8.65 MAU: 56.00 ± 13.00 Controls: 50.80 ± 8.05	NAU: 102.02 ± 17.80 MAU: 97.87 ± 8.83 Controls: 103.65 ± 11.9	3	NAU: 16.43 ± 2.12 MAU: 17.03 ± 1.85 Controls: 16.66 ± 1.63	NAU: 34.61 ± 3.39 MAU: 28.14 ± 3.76 Controls: 28.85 ± 3.76	NA	BOLD imaging can provide a noninvasive assessment of hypoxia in the early stage of diabetic nephropathy with a significantly higher R2* value in the renal medulla in diabetic patients without proteinuria, indicative of medullary hypoxia
*Chen, Kidney Blood Press Res* (2021) [109]	Compare renal tissue oxygenation using two BOLD MRI analysis methods	*n* = 50, including 40 CKD and 10 controls	CKD: 44.2 ± 14.9 Controls: 43.2 ± 14.4	CKD: 42.44 ± 32.86 Controls: 110.86 ± 12.78	3	CKD: 20.2 + 3.0 Controls: 17.2 + 1.4	Controls: 23.6 + 0.9 CKD: 31.8 + 3.2	Controls: 26.2 + 1.2 CKD: 34.3 + 3.4	Cortical and medullary oxygenation were significantly reduced in patients with CKD compared with healthy subjects. Whole kidney R2* values were correlated with GFR and renal plasma flow
Laursen, *Clin Kidney J* (2022) [154]	Investigate kidney oxygenation in adults with type 1 diabetes and diabetic kidney disease	*n* = 30, including 15 T1D and 15 controls	T1D: 58 ± 14 Controls 56 ± 15	T1D: 73 ± 32 Controls: 88 ± 15	3	T1D: 22 ± 5 Controls: 22 ± 3	T1D: 34 ± 6 Controls: 38 ± 5	NA	Participants with T1D and albuminuria exhibited higher medullary oxygenation than controls. Lower cortical oxygenation was associated with higher proteinuria and lower GFR in T1D
Seah, *J Diabetes Complications* (2022) [59]	Compare levels of renal hypoxia measured by BOLD MRI in patients with T1D and controls	*n* = 42, including 34 T1D and 10 controls	T1D: 45 (32–55) Controls 45 (31–55)	T1D: 105 (77–120) Controls: 94 (80,99)	3	T1D: 14.7 (13.7–15.8) Controls: 15.7 (15.1–16.6)	T1D: 24.8 (21.8–28.2) Controls: 29.3 (24.3–32.4)	NA	Reduced cortical and medullary R2* in type 1 diabetes, reflecting more oxygenated parenchyma, compared to healthy controls; and no statistically significant differences between participants with type 1 diabetes that were hyperfiltering and non-hyperfiltering
Sørensen, *NDT* (2023) [155]	Investigated whether renal oxygenation measured with BOLD MRI differs between people with type T2D with normal to moderate CKD (Stages 1–3A) and matched controls	*n* = 40, including 20 T2D and 20 controls	T2D: 69.2 ± 4.7 Controls: 68.8 ± 5.4	T2D: 86.9 ± 18.7 Controls: 86.1 ± 18.5	3	T2D: 19.54 (19.10–19.9) Controls: 20.08 (19.63–20.54)	T2D: 22.75 (22.38–23.11) Controls: 23.40 (23.01–23.79)	NA	T2D patients with normal to moderate CKD do not seem to have lower renal oxygenation when measured with BOLD MRI
Prasad, *KI Rep* (2023) [114]	Evaluate the association between the BOLD signal and fractionated blood volume	*n* = 13, including 6 CKD diabetic patients and 7 controls	CKD: 68.4 ± 6.6 Controls: 40.1 ± 15.3	CKD: 31.7 ± 13.3 Controls: 97.9 ± 15.7	3	CKD: 18.88 ± 2.92 Controls: 17.64 ± 1.00	CKD: 24.36 ± 3.49 Controls: 26.64 ± 3.37	NA	Taking fractionated blood volume into account, cortex is normoxemic in healthy subjects and moderately hypoxemic in CKD; medulla is mildly hypoxemic in healthy subjects and moderately hypoxemic in CKD

NA, non-applicable; T2D, Type 2 diabetes; T1D, Type 1 diabetes; HBP, high blood pressure; MAU, moderately increased albuminuria; NAU, normal to mildly increased albuminuria; data are expressed as mean ± standard deviation or median (interquartile range).

#### Evaluation of pharmacological nephroprotective and nephrotoxic effects

In albuminuric diabetic patients, a single dose of SGLT2 inhibitor (SGLT2i) Dapagliflozin reduced cortical R2* after 6 hours compared with placebo, without perfusion modification [119]. Similarly, a reduction in cortical and medullary R2* was observed after 24 weeks of treatment with Canagliflozin in type 2 diabetic patients without CKD [120]. These results suggest improved renal oxygenation with these SGLT2i, which may partly explain their nephroprotective effect [121]. Surprisingly, no acute or sustained changes were observed in cortical or medullary oxygenation measured with BOLD MRI in healthy subjects treated with Empagliflozin [122]. The mechanism of this observation remains unknown, one hypothesis mentioned by the authors is a shift of oxygen-consuming active transport to the medulla. Further studies are needed to determine the implication of confounding factors such as glycemia, hyperfiltration, proteinuria, or CKD on the BOLD signal after treatment with SGLT2i. GLP1 analogues also preserved cortical and medullary R2* after salt loading in healthy subjects, in favor of preserved tissue oxygenation potentially contributing to the renal and cardiovascular benefits of these drugs [123]. On the other hand, BOLD MRI can help in evaluation of pharmacological nephrotoxic effects which are associated with reduced renal oxygenation. In a rodent model, BOLD MRI could detect proximal tubular toxicity of aminosides after Gentamycin injection [124]. This sequence also enables early detection of iodine contrast-induced nephropathy in animals, for which renal hypoxia is recognized as a key pathophysiological factor [125, 126]. BOLD MRI appears to be a promising tool to assess nephroprotective and nephrotoxic effects of drugs in development.

## MAGNETIC RESONANCE RELAXOMETRY (T1 AND T2 MAPPING)

### Biophysical and technical principles

**Figure 3:**
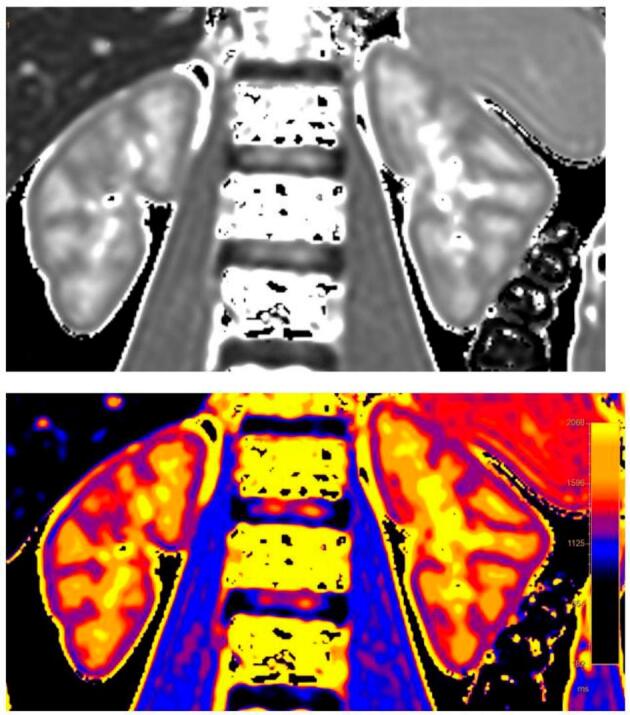
Coronal T1 mapping representative image in a 47-year-old healthy woman showing the corticomedullary differentiation. Upper: T1 map, lower: T1 map colorized. T1 values are higher (orange) in the medulla than in the cortex (purple), due to higher mobility of water molecules in the tubules and collecting ducts. Acquisition parameters: Voxel volume 3 × 3.03 × 6; repetition time: 2.5 ms; echo time: 1.2 ms; Matrix: 112 × 98; Field of view 330 × 296; acquisition time: 11 s.

Magnetic resonance relaxometry is used routinely in cardiology for the diagnosis of cardiac amyloidosis, Fabry disease, or myocarditis, whereas its use in nephrology remains almost exclusively limited to research purposes [8]. A magnetic field B0 is applied, and the magnetic moments of the protons orient themselves in the same direction as the magnetic field. After excitation of the protons by a millisecond and milli-Tesla radiofrequency pulse called B1, the protons return to the axis of B0. This relaxation is accompanied by energy emission in the form of radiofrequency waves, which constitutes the signal recorded by nuclear magnetic resonance. T1 is the time required for longitudinal magnetization to reach 63% of maximum longitudinal relaxation and T2 is the time required for transverse magnetization to reach 37% of maximum transverse relaxation. These relaxation times are characteristic of tissue composition. In healthy subjects, T1 and T2 are higher in the medulla than in the cortex, presumably due to a higher free water content in the tubules (Fig. 3) [127]. Various acquisition methods are available for T1 mapping [8, 13]. The gold standard is the inversion recovery method: a first radiofrequency pulse at 180° reverses the direction of the magnetic field, followed by a waiting time called inversion time (IT), and then, by pulses at 90° and 180°. This method must be repeated several times with increasing IT.

Other acquisition techniques have been developed to shorten acquisition times: the look-locker sequence and its variants such as modified look-locker inversion recovery, and variable flip angle (VFA). The look-locker sequence and its variants are recommended and enable T1 measurement in a single breath-hold. They consist of repeated fast gradient-echo pulses with increasing IT after a single 180° inversion pulse. The VFA method does not use a 180° inversion pulse, the flip angle of the radiofrequency pulses is variable, and is currently not recommended for renal imaging [13]. Other methods are being evaluated [128].

For T2 mapping, the most used acquisition method is the multi-echo spin-echo sequence, which consists of a 90° radiofrequency pulse followed by several 180° radiofrequency pulses [8, 13]. The alternative technique is the gradient and spin-echo method using both spin-echo and gradient-echo characteristics. T1 and T2 are influenced by magnetic field, with higher T1 values, larger T1 corticomedullary difference, and a decrease in T2 values at 3.0 T than at 1.5 T [129]. T2 is also influenced by scattering and slice profile ,which may be counteracted by preparation modules. Preparation modules have been validated and compared for cardiac imaging, but not for renal imaging [130, 131]. Recovery time is essential because incomplete T1 recovery can lead to errors in T2 measurements [132]. Comparison of these different schemes requires validation using phantoms in the T1 and T2 *in vivo* reference ranges. Such phantoms have been used to harmonize brain and heart studies, but few data exist renal MRI [133].

### Applications

#### Chronic kidney disease

Higher cortical T1 values and corticomedullary dedifferentiation have been observed in CKD patients. GFR negatively correlate with cortical T1 and corticomedullary differentiation [38, 129, 134–138]. Among the few studies that have included histological analysis on native kidney, most found an association between IF percentage and corticomedullary dedifferentiation [136]. Wei *et al.* suggested, based on a cohort of 71 CKD patients, a corticomedullary T1 difference of −343.81 ms and a cortex/medulla T1 ratio of 0.8359 as optimal cutoff values for differentiating severe (>50%) from mild-moderate (25–50%) IF with high sensitivity (both 100%) and specificity (respectively, 90.6% and 94.3%) [139]. Combination with DWI MRI seemed to improve specificity for fibrosis assessment [37, 40, 38, 39]. However, one study comparing 20 IgA nephropathy patients with 10 healthy subjects found no association between histological data, especially histological S score (presence of segmental glomerulosclerosis) from the Oxford classification/MEST score, and T1 values [138, 140]. Such discordance may be explained by the fact that T1 is influenced by many factors like comorbidities, acquisition methods, and patient preparation. Moreover, corticomedullary ADC difference was better correlated to IF than corticomedullary T1 difference in 164 patients undergoing renal biopsy [37]. T1 mapping seems to be more sensitive than specific for fibrosis detection, and its place and specificities compared to DWI need to be clarified.

T2 mapping appears to be useful in the setting of autosomal dominant polycystic kidney disease (ADPKD). In ADPKD animal models, T2 values allowed an early detection of the disease, at stages when total kidney volume was not increased [128]. These results have been confirmed in humans, with a strong association between parencymal T2 values and disease severity (percentage of cysts) [141].

#### Acute kidney injury

Like DWI, T1 mapping might detect AKI in an evolving process. Indeed, in a murine model of unilateral moderate (35 min) and severe (45 min) IRI, T1 values significantly increased until day 7, and correlated with AKI severity. The largest T1 changes were observed in the outer stripe of the medulla, corresponding to cell swelling, capillary leakage, and interstitial edema in this area highly susceptible to hypoperfusion and hypoxemia [142]. On day 28, while T1 values reached baseline in moderate IRI, they remained high in severe IRI group, consistent with complete tubular regeneration after moderate IRI, and edema, cell swelling, and inadequate tubular repair with IF in severe IRI group. In nine severe AKI patients, Buchanan *et al.* found increased cortical and medullary T1 with decreased corticomedullary differentiation [143]. After 1 year of follow-up, as the AKI healed, T1 values decreased and gradually returned to baseline consistent with resolution of edema and inflammation. However, in some participants, cortical and/or medullary T1 remained high at 1 year possibly reflecting subclinical chronic lesions. The clinical implications and prognosis of these persistent alterations remain unclear and need to be addressed. In another study, T1 mapping was performed within 2 weeks after solid organ transplantation (49 kidney transplantation, 52 lung transplantation), with an increase in T1 after solid organ transplant [144]. This increase was more pronounced following kidney transplantation, was associated with AKI severity, correlated significantly with renal function, and might correspond to edema and inflammation following the surgery.

Data on the significance of T2 in renal imaging is limited. T2 seems to allow characterizing tissue water content [145]. In a 45 min IRI rat model, a global decrease in T2 during the ischemia period was observed, with T2 enhancement in the inner medulla and cortex during the immediate reperfusion period, but not in the outer medulla consistent with tubular necrosis and with persistent hypoperfusion that resulted in endothelial cell injury and edema [146]. Changes of T2 evaluated 1 week after reperfusion were associated with the severity of AKI, as well as IF at 1 month [68]. By contrast, in a 60 min IRI rabbit model with different MRI evaluation timing, an increase in T2 in the outer medulla was observed after 1 h, followed by a reduction for up to 48 hours, which correlated to tubular epithelial edema but not with tubular necrosis, inflammation, and cast [147]. In a sepsis-associated AKI rat model, Zhao *et al.* showed a reduced cortical and medullary T2 relaxation time at 18 h with a decrease in cortical perfusion, which could predict survival outcomes at 96 hours [148]. Since T2 is sensitive to paramagnetic effect of deoxyhemoglobin, this observation supported the fact that low T2 values may also reflect hypoxia [149]. To our knowledge, no study has evaluated T2 in the setting of AKI in human.

## CONCLUSION

Multiparametric MRI has the potential to provide a better understanding of renal physiology and pathophysiology, a better characterization of renal lesions, an earlier and more sensitive detection of renal disease, and an aid to personalized patient-centered therapeutic decision-making. The development of these noninvasive tools offers numerous possibilities for the diagnosis, prognosis, assessment of predictive factors, and follow-up of kidney disease, from preclinical evaluation of treatments to direct translation to human disease and future best-practice care. Further data and clinical trials are needed to allow its routine application in clinical practice.

## Data Availability

No new data were generated or analyzed in support of this research.
